# Supplementary material for: External Globus Pallidus Arkypallidal Circuit Dynamics Gate Risk-Taking Behavior
Source: bioRxiv. 2026 Mar 23:2026.03.20.713182. Preprint. [Version 1] doi: 10.64898/2026.03.20.713182 (PMC13041812; doi:10.64898/2026.03.20.713182)
Supplement: Supplement 1 [file media-1.pdf]

Supplementary Data 1 - Representative Closed-to-Closed transition in the elevated plus maze.
Representative overhead video of a mouse performing a Closed-to-Closed transition in the elevated plus maze. Colored pose-estimation keypoints are overlaid on the animal throughout the sequence. Video is shown at real speed (30 frames/s). The clip illustrates movement from one closed arm, through the center, and into the opposite closed arm without open-arm commitment.

Supplementary Data 2 - Representative NoGo sequence in the elevated plus maze.
Representative overhead video of a mouse performing a NoGo sequence in the elevated plus maze. Colored pose-estimation keypoints are overlaid on the animal throughout the sequence. Video is shown at real speed (30 frames/s). The clip illustrates approach toward the center and open-arm junction without subsequent commitment to open-arm exploration.

Supplementary Data 3 - Representative Go sequence in the elevated plus maze.
Representative overhead video of a mouse performing a Go sequence in the elevated plus maze. Colored pose-estimation keypoints are overlaid on the animal throughout the sequence. Video is shown at real speed (30 frames/s). The clip illustrates risk assessment followed by forward commitment into the open arm.

Supplementary Data 4 - Representative Closed-to-Open transition in the elevated plus maze.
Representative overhead video of a mouse performing a Closed-to-Open transition in the elevated plus maze. Colored pose-estimation keypoints are overlaid on the animal throughout the sequence. Video is shown at real speed (30 frames/s). The clip illustrates traversal through the center followed by commitment to an open arm.
